# “The Key Is to Value Every Little Achievement”: A Qualitative Study of the Psychological Experience of Parent Caregivers in Paediatric Palliative Care

**DOI:** 10.3390/clinpract13030062

**Published:** 2023-06-06

**Authors:** Alexandra Jóni Nogueira, Maria Teresa Ribeiro

**Affiliations:** CICPSI, Faculty of Psychology, University of Lisbon, 1649-013 Lisbon, Portugal; mteresaribeiro@psicologia.ulisboa.pt

**Keywords:** family, experience, chronic illness, health psychology, qualitative methods

## Abstract

In Europe, Portugal has been identified as the country with the most rapid evolution of paediatric palliative care provision, which is a highly complex experience for families. The present descriptive–exploratory study seeks to contribute to the understanding of the psychological experience of life-limiting conditions in parent caregivers. A total of 14 families completed a sociodemographic and clinical data sheet and answered a structured online interview based on an incomplete narrative resulting from the Unwanted Guest Metaphor. A thematic analysis of the various narratives was performed through an inductive–deductive process. The results provide a holistic view of 10 essential dimensions in the parental psychological experience and contribute to the design of intervention methodologies in an eco-systemic approach. The importance of clear communication with health professionals, an awareness of the unpredictability of the disease, the desire for more self-care, the difficulty in understanding their children’s needs and the threat implicit in everyday life are some of the main findings. This research emphasizes the importance of having opportunities of emotional expression and psychoeducation about anxiety’ management, enhancing the perception of positive characteristics in children with palliative needs and creating time for the couple. The study has some limitations, such as the small sample size, and suggests that further research should explore the father’s experience.

## 1. Introduction

Palliative care refers to “holistic and proactive care for children, adolescents and adults who will not get better from their illness” [1]. It improves the quality of life of patients and that of their families who are facing challenges associated with life-threatening illness, whether physical, psychological, social, or spiritual [2]. People with palliative needs must have a complex chronic disease affecting a specific organ or different systems, is sufficiently severe, that has lasted for at least 12 months, and that requires specialized care [3].

Palliative care uses a multidisciplinary team approach to prevent and relieve suffering through early identification, correct assessment, and the treatment of pain and other problems. Palliative care should be offered once the diagnosis of the disease has been made, accompanying the acute, chronic, and terminal phases of the illness, as well as providing bereavement counselling to families, though it is regularly confused with end-of-life care [2,4].

In this context, paediatric palliative care is active care for children and adolescents with life-limiting or life-threatening conditions, regardless of their diagnosis or stage of illness [5]. It constitutes a basic human right and has been recognized by the World Health Organization since 1988, wherein every child should expect individualised palliative care that is appropriate to their culture and age, regardless of their age, diagnosis, residence, culture, or socioeconomic status [4,6]. The development of paediatric palliative care, classically seen as an active approach that encompasses the entire process of disease diagnosis, throughout the life cycle and up to the post-mortem period, should be seen as a public health and human rights priority. Its main goal is to promote the child’s physical, psychological and spiritual well-being, while also ensuring the necessary support for the whole family, and providing an improvement in the quality of life of all those involved [6].

According to information provided by the International Children’s Palliative Care Network, the provision of this care has expanded on a worldwide scale, with countries such as Australia, Canada, Germany, Belarus, the United Kingdom, the Netherlands and the United States of America being at level five (of five), i.e., with evidence of broad palliative care provision for children, based on a comprehensive approach of full integration in the health services as well as national policies to support children’s palliative care. For its part, Portugal has shown evidence of faster progress in providing this care at a European level. It has been positioned at level four (of five) since 2018 due to its broad provision of palliative care for children and its offer of training and focused plans for the development of services and integration in health care services [7]. In 2018, an estimated 7828 children/youths were identified as having palliative needs [8].

In order to operationalize the multiple situations that may benefit from paediatric palliative care, all families with a child or adolescent diagnosed with a life-limiting illness that may fall into one of the four categories defined in the literature are considered [9]: Category I corresponds to life-threatening conditions for which curative treatment may be feasible but can fail; Category II refers to the conditions under which premature death is inevitable, potentially involving long periods of intensive illness-oriented treatment; Category III corresponds to progressive conditions without curative treatment options; and Category IV refers to irreversible but non-progressive conditions causing severe disability. Some examples of diseases are cancer, cystic fibrosis, Duchenne muscular dystrophy, metabolic conditions, and severe cerebral palsy.

The experience of a potentially fatal illness process has a significant impact on the whole family structure and dynamics, given the complex nature of family life. It is understood as a stressful life event that influences the processes of the emotional, social and spiritual development of children and their families, as well as the psychological adaptation of the individual and the family to the illness context [10]. It is therefore essential to understand the impact of the illness on the various members of the family system through a reflection on their psychological experience in the context of such illness [11], aligning their needs to the intervention provided by the health professionals within an ecosystemic approach [12]. The psychological experience is understood as the set of emotions, thoughts and behaviours originating from and/or experienced within the context of the illness.

### The Psychological Experience of Parent Caregivers

Parents play a central role as the primary caregivers, legal guardians and advocates of their medically complex child [13]. However, most families in paediatric palliative care settings are weakened in their socioeconomic situation, because only one caregiver assumes the responsibility of financially supporting the family [14]. As a rule, it is the mother who becomes the primary caregiver while the father maintains or doubles his work activity [15,16]. This reality may also explain why the literature has focused primarily on the impact for the mother caregiver [17,18], although this study aims to include mothers and fathers. A study regarding fathers highlighted their challenges when living with such an illness on a daily basis and their emotional struggles [19].

The literature has reinforced the impact of this caregiving experience on well-being and quality of life. Nogueira and Francisco concluded that parents manifest fatigue and stress in the management of daily tasks, have constant concerns and feel satisfaction with the sick child’s achievements [20]. The studies highlight the psychological distress of the parental subsystem and refer to several predictor variables, such as the role of the child/youth’s age and the caregiver’s level of schooling [21]. A study with 264 parents of children with cancer in Jordan also found that 75.4% of them manifested mild-to-severe levels of burden. The highest level of burden is predicted by factors such as financial difficulties, progression of the illness and significant anxiety and depressive symptomatology [22]. The characteristics of caregivers and those being cared for, the family itself and health services also contribute to this challenge [23].

Several international studies have identified communication difficulties, intellectual disability and the child/youth’s pain as potential stressors in caregivers [24,25]. According to the literature, the time of diagnosis of the illness is also associated with the symptomatology related to post-traumatic stress [26]. In fact, parents of children with life-limiting conditions associate greater stress with care, hospitalizations, readmissions to health care [27,28] and their multidimensional roles [29]. The dyadic coping in the parental subsystem is important [30] because the characteristics and motivations for co-parenting undergo changes throughout the evolution of the illness [31]. Higher parental self-efficacy predicts maternal psychological well-being 3 months after the child’s discharge [32,33].

Parent caregivers tend to manifest daily anxiety and stress as a result of confronting the potential loss of the child, preservation of a close and reassuring relationship with the child and tension related to end-of-life decisions [34,35]. In fact, part of this new normal is the acceptance of a life characterized by chronic uncertainty, specifically regarding the future [36,37]. The literature also highlights guilt over decreased attention to healthy children [20,38].

With regard to their coping strategies, some of the literature highlights how parents resort to social support, including seeking informational and emotional support through interactions on social media with families in similar circumstances [39,40,41]. Other strategies can be emotional expression and optimism [42], leisure activities, information seeking, good family cooperation, spiritual support and faith [35,43], mobilization of family resources, attempts to maintain normality in daily life, live one day at a time, distraction from the illness and relativization [36]. Regarding their needs, parents emphasize the importance of receiving more support, having time for themselves (70%), knowing what they can expect in the future (64%) and receiving adequate information to understand the illness (39%) [35]. It is essential that family members are able to develop an attitude of empowerment, resilience and coping in the face of this situation.

There is a consensus that the experience of a paediatric life-limiting condition is traumatic for children and their family [26,44]. Following it, parent caregivers can experience post-traumatic growth [45], and benefit finding is the process by which the parents assign positive value to how the adversity affected them in that moment of their lives [46,47].

The literature suggests that parent caregivers are an at-risk population, reinforcing the relevance of studying their experiences from their own perspective [48]. Additionally, it should be noted that, to date, most of the literature has focused on oncological diseases, giving little attention to neurological, renal, cardiovascular, congenital, and haematological diseases, among others. Therefore, this study seeks to understand the experience of the parental subsystem—with mothers and fathers—considering the heterogeneity of existing complex chronic diseases, thus filling a gap in the scientific research.

Based on these considerations and based on a descriptive–exploratory approach, the aim of this study is to contribute to an understanding of the psychological experience of life-limiting conditions in parent caregivers through their own perspective. Therefore, the guiding research question of this study is as follows: “How do parent caregivers psychologically experience a life-limiting condition?”.

As specific objectives, defined upon the conclusion of the literature review, this study explores the parent caregivers’ psychological experience in the context of paediatric palliative care, namely: confrontation with the diagnosis; representation of the illness; emotional impact; day-to-day challenges; family impact; resources and social support; coping strategies; post-traumatic growth; representation of the sick child; and future perspectives.

By exploring the specific features of parent caregivers’ psychological experience, this research allows for the identification of concrete needs, resulting in the development of skills and the activation of key processes to foster family resilience and post-traumatic growth in this population. This perspective is consistent with the definition of national strategic guidelines for Palliative Care in 2021–2022—namely, in focusing care on the person, (re)integration of the family and strengthening the social network through the identification of family needs, the promotion of their adaptation to the illness, the preservation of autonomy and the psychosocial support [49]. Finally, it contributes to the development of systemic psychological intervention in paediatric palliative care, both in terms of guidelines and previously developed and assessed methodologies.

## 2. Materials and Methods

### 2.1. Procedure

This study uses a qualitative, descriptive–exploratory and cross-sectional design. Following the assessment of the formal ethics review committees by the Ethical and Deontological Committee of the Faculty of Psychology of the University of Lisbon, a pilot study was conducted with three parents in order to assess the need for possible changes to the initially proposed protocol. Changes were made to four questions, making them more creative and minimizing the risk of bias, since the words initially chosen had a dubious interpretation. In addition, the reworded questions made it possible to obtain more comprehensive and complete answers.

The evaluation protocol used in this study was then made available online through the GoogleDocs platform. This was disseminated on social networks, and a non-probabilistic method—namely, snowball and convenience sampling—was adopted. Simultaneously, several national institutions were contacted in order to present this research and call for its dissemination among potential participants. A recruitment of at least 12 parent caregivers was expected.

The inclusion and exclusion criteria were defined for the recruitment and selection of the sample. Thus, to participate in this study, all parents had to be at least 18 years of age, be the mother or father of a child or youth with a life-limiting condition—covered by one of the four categories identified in the literature [9]—diagnosed for at least 12 months and living with the child or youth. In addition, the child/youth with the condition had to be 18 years of age or younger. Having more than one child with a complex chronic illness and not being able to read and/or write in Portuguese were considered exclusion criteria. There was no control group in this study. Informed consent was obtained from all subjects involved in the study, which was available on the platform, and then the subjects voluntarily and anonymously responded to this study.

Finally, a thematic analysis [50] of the various narratives was performed through an inductive–deductive process, which allowed for the comparison and integration of the results. Despite this being a recursive process, the researchers initially familiarized themselves with the information written by the participants and made note of the ideas related to themes that could become categories. Later, and in a systematic manner, all the answers were coded, placing the relevant data in each defined category and subcategory. The unit of analysis was mostly the sentence; however, there was flexibility to code parts of a sentence or a whole paragraph whenever deemed necessary for a particular category. The identification of themes occurred mainly at the latent level, in order to identifying the underlying ideas and conceptualizations. Similarly, the thematic analysis was inductive, since the identified themes were strongly related to the explicit data in the participants’ responses.

### 2.2. Participants

#### 2.2.1. Parent Caregivers

The sample of this study consists of a total of 14 families of children in paediatric palliative care, with 13 mothers and 1 father. They are aged between 27 and 48 years (M = 39.79, SD = 5.99) and all have Portuguese nationality. Regarding marital status, nine (64.3%) are married, three (21.4%) are cohabiting, one (7.1%) is single and one (7.1%) is separated. Regarding academic qualifications, most of them have completed 12th grade (*n* = 6) and hold a Bachelor’s degree (*n* = 3). As for their current employment situation, six (42.9%) are employed, four (28.6%) are on sick leave, three (21.4%) are unemployed and one (7.1%) is a student. Of the 14 participants, 5 (35.7%) have used mental health services in the past and 3 (21.4%) currently benefit from these services.

#### 2.2.2. Children with Life-Limiting Conditions

Nine of the children are male (64.3%) and five are female (35.7%), with ages ranging from 2 to 15 years (M = 8.79, SD = 4.04). All the diagnostic categories in paediatric palliative care [9] are represented in this study, with particular emphasis on some illnesses, such as cerebral palsy, chromosomopathy, osteogenesis imperfecta, moderate–severe haemophilia, Rett Syndrome and polymalformative syndrome. Of these children, half are dependent on medical respiratory technology; six (42.9%) were diagnosed with the disease up to seven years ago, and eight (57.1%) up to eleven years ago. All were subjected to hospitalizations, with 8 (57.1%) being hospitalized 1 to 9 times, 2 (14.3%) being hospitalized 10 to 19 times, 3 (21.4%) being hospitalized 30 to 39 times and 1 (7.1%) being hospitalized more than 50 times. As for the child/youth’s level of disability, 5 (35.7%) have between 60 and 89%, 5 (35.7%) between 90 and 99% and 4 do not know or did not answer. Most of the children and youths (*n* = 11) currently benefit from institutional support. It is also noteworthy that in 11 of the participating families (78.6%), the mother is considered the main caregiver, and in 3 (21.4%), the father and mother are equally considered caregivers. As for knowledge about paediatric palliative care, 11 of the parent caregivers (78.6%) are not familiar with this service, 3 (21.4%) have heard about it, but only 1 of them has benefited from this type of care.

#### 2.2.3. Family Household

The household is composed of two to five members (M = 3.71, SD = 0.99); in nine families (64.3%), the father is part of the household, and in ten families (71.5%), there are healthy siblings. Among these, 2 (14.3%) are under the age of 1 year, 1 (7.1%) is aged between 1 and 5 years, 2 (14.3%) are between 6 and 10 years, 4 (28.6%) are aged between 11 and 15 years and 1 (7.1%) is over 20 years of age.

### 2.3. Instruments

The protocol for assessing the psychological experience of illness by parent caregivers consists of a sociodemographic and clinical data sheet and an online structured interview script, presented below.

#### 2.3.1. Sociodemographic and Clinical Data Sheet

The aim of this sheet is to collect information to characterize the study sample—namely, data regarding the parents (e.g., age, gender, marital status, employment status, use of mental health services), the child/youth with a life-limiting illness (e.g., age, illness diagnosis, technological dependence, hospitalizations, current institutional support) and the family (e.g., household composition, age, school attendance/professional occupation) [51].

#### 2.3.2. Online Structured Interview Script

This script followed an incomplete narrative based on the Unwanted Guest metaphor, also known as the Joe-the-Bum metaphor, used in acceptance and commitment therapy [52]. The dimensions under assessment correspond to the specific features of the illness adaptation process of this population, and were selected following the literature review, namely: confrontation with the diagnosis; representation of the illness; emotional impact; day-to-day challenges; family impact; resources and social support; coping strategies; post-traumatic growth; representation of the sick child; and future perspectives. This script was constructed on the basis of narrative therapy strategies [53], promoting conversational interaction between the participant and the researcher, through an online written platform. Thus, some guiding questions are asked and the content of each participant’s answers reflects their dominant narrative about the way they experience the illness [54], such as: “If I asked your child’s illness to tell me about you, what do you think it would tell me?” and “If you could talk to them [other parents] and give them three of your ‘tricks’ to help them live better with the illness, what would you tell them?”. An average interview time was estimated to be 25 min.

### 2.4. Method of Data Analysis

The categories were organized into potential higher-level categories, aiming to construct an initial thematic map. This process resulted in a review of the themes in relation to the quotes that had been coded and the data overall, making it possible to refine the previously defined categories. In some cases, the categories were renamed, and changes were made to the initial coding of the excerpts. This step-by-step process culminated in the completion of a coherent and detailed system of categories, allowing for the initially defined research question to be answered.

The data from 14 in-depth interviews was analysed in the order in which they were conducted and there was a relatively homogeneous study population and narrowly defined objectives. To assess saturation, we used code meaning, which focused on reaching a full understanding of issues in data as the indicator that saturation is reached, by assessing whether the issue, its dimensions, and nuances are fully identified and understood. All the interviews were reviewed, one by one, and saturation was reached at the point at which little or no relevant new codes and categories were found in the data and when issues started to repeat themselves with no further understanding or contribution to the study phenomenon and its dimensions.

For this analysis, NVIVO^®^ 12 software (QSR International, Burlington, MA, USA) was used, which enables better organization of the data through the category tree. For the sociodemographic characterization of the families, SPSS^®^ 26 statistical software (IBM, Armonk, NY, USA) was used.

## 3. Results

**Figure 1:**
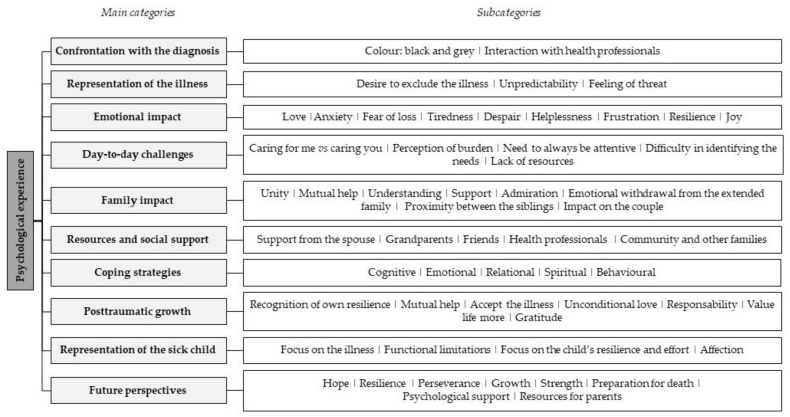
Summary of the results based on the main categories and subcategories.

The data analysis resulted in a system of categories, with a total of 156 items, of which 10 were the main categories aimed to meet the specific objectives of this study (Figure 1).

### 3.1. Confrontation with the Diagnosis

When assigning a colour to represent the moment of diagnosis, five of the parent caregivers chose black, justifying this as follows: “I feel I’m in darkness with no north or south, without light, everything I try to reach flees from my hands” (X., mother of a six-year-old child with congenital glycosylation disorder). In turn, three participants chose grey and three chose red, explaining “On alert, having to intervene quickly, to act, to react, to fight” (M., mother of a 10-year-old child with chromosomopathy). Four of the parent caregivers reported feeling helpless when confronted with an unknown reality, and three reported psychological suffering associated with the threat to life and happiness.

On the other hand, interaction with the health professionals was also mentioned by the participants:

I would like to have received a hug at the diagnosis, to feel 100% honesty when they talked to us, what happened is that they frequently omitted things that obviously must have been known because of the sequelae, I would like to feel that they were 100% transparent. (S., mother of a nine-year-old child with heart disease, severe lung disease, stroke and anaphylaxis).

### 3.2. Representation of the Illness

In this study, most of the parents reinforced the desire to exclude the illness from their lives: “Go away and let my little boy play and be able to ‘hurt himself’ without the worry of it turning into something serious” (X., mother of a two-year-old child with moderate–severe haemophilia). In addition, six of the participants mentioned the unpredictability of the illness as an important factor in the loss of control, and five parents emphasized the feeling of threat and risk inherent to the disease, referring to their perception of the risk involved:

That our inability to cure him consumes our energy and peace of mind and takes away our sleep, that what is so simple and guaranteed for many, is not given, acquired at birth, it is fought for, aspired to and desired by the special ones. (X., mother of a six-year-old child with congenital glycosylation disorder).

### 3.3. Emotional Impact

Eight participants referred to love as the main emotion they felt towards their children with a complex chronic disease and half of the sample reported anxiety as the central emotion in their daily lives. On the other hand, fear, particularly associated with loss, was also described by six of the parent caregivers, as sadness. The expression of tiredness, despair, helplessness and frustration was also highlighted:

You know, since my daughter was born and I discovered her illness, I have lost my freedom for everything from outings to jobs. I live for her and live like a hostage, I feel like a beggar, I’m tired, I see my youth slipping away and my retirement too. (X., mother of a 14-year-old child with Rett Syndrome).

Additionally, and by using positive emotions, three parents mentioned resilience and three parents mentioned joy in their daily lives.

### 3.4. Day-to-Day Challenges

All the parent caregivers mentioned the challenge of caring for me vs. caring for you as essential in their daily lives: “That you need to rest so that you can continue caring” (M., mother of a 10-year-old child with chromosomopathy). In this sense, when asked about their perception of burden on a scale of 1 to 10, most participants attributed a score of 10, 9 and 8. In fact, 6 of the 14 parent caregivers highlighted the demands of caregiving on their life and family.

Regarding the relationship with their sick children, half of the parents highlighted the need to always be attentive and the difficulty in identifying the needs of the children/youths:

Difficulty understanding my child, they wouldn’t know when he would be hungry, thirsty or have a dirty diaper, he would have a fit and they would smile at him without realizing it, or even worse, not knowing what to do, if he cried they wouldn’t know if he was uncomfortable, in pain or having a tantrum. (X., mother of a six-year-old child with congenital glycosylation disorder).

Half of the sample identified the lack of resources for these families: “My son needs to have the same opportunities as any other child” (S., mother of a four-year-old child with severe diffuse congenital hyperinsulinism).

### 3.5. Family Impact

Most of the participants identified changes in family resilience skills, emphasizing unity, mutual help, understanding, support and admiration in the family system. Furthermore, eight of the parent caregivers reported an emotional withdrawal from the extended family. Within the nuclear family itself, it should be noted that a mother mentioned proximity between the siblings, i.e., between the sick child and the healthy children, as a positive change following the diagnosis of the illness.

The participants also highlighted the importance of the impact on the couple: “Little time for the couple to spend together” (N., father of a sick 11-year-old child without an etiological diagnosis).

### 3.6. Resources and Social Support

The participants reinforced their own family, specifically support from the spouse and the grandparents: “Grandparents for fighting for their grandchild and finding conditions to support so many expenses, although they are still tired” (X., mother of a six-year-old child with congenital glycosylation disorder).

According to half of the sample, friends were also a source of support, and they also emphasized the health professionals: “The medical team currently accompanying my son is top!!! They only do not do what they are unable to. They are united in saving my son” (S., mother of a nine-year-old child with heart disease, severe lung disease, stroke and anaphylaxis).

Support from the community and other families was also mentioned: “Other parents and relatives of children with the same pathology, for the support and sharing” (S., mother of a four-year-old child with severe diffuse congenital hyperinsulinism).

### 3.7. Coping Strategies

In regard to cognitive strategies, 9 of the 14 participants identified continuous learning, not suffering in anticipation, actively seeking solutions—“That I don’t leave the illness alone, always looking for solutions” (T., mother of a 3-year-old child with osteogenesis imperfecta and Ehlers–Danlos Syndrome)—relativizing and allowing time for preparation.

On an emotional level, the most frequent strategies were being perseverant, managing hope—“I didn’t choose a potentially fatal illness for my child, but I choose what to do with it. And as far as it’s up to me it will be something positive” (S., mother of a nine-year-old child with heart disease, severe lung disease, stroke and anaphylaxis)—having patience and courage, being calm and knowing that the illness is not a child/youth’s characteristic. At the relational level, the participants tended to ask for help, to share their experience with other families in similar circumstances, to encourage the child/youth and to trust the health professionals. Spiritually, they tried to promote their faith.

The behavioural strategies most adopted by the participants were resting from the illness, normalizing, living one day at a time and valuing the small achievements: “That the key is to value every little achievement and enjoy every smile, always” (M., mother of a 10-year-old child with chromosomopathy).

### 3.8. Post-Traumatic Growth

Half of the sample reported recognition of their own resilience and the importance of mutual help, especially at the intra-family level, was also mentioned. Additionally mentioned were the ability to accept the illness and its impact and the unconditional love felt towards their children. Other participants highlighted the fact that they felt they had matured as people, becoming more responsible, that they valued life more and felt gratitude and were more sensitive to the needs of others: “I often offer help because I know it is hard to ask for it” (M., mother of a 10-year-old child with chromosomopathy).

### 3.9. Representation of the Sick Child

Most of the participants focused on the illness, emphasizing its specific features, its impact and the resulting functional limitations: “My son can’t walk, sit, talk, he needs me for everything and I need to work to support him” (X., mother of a six-year-old child with congenital glycosylation disorder). Parent caregivers also maintained a focus on the child/youth’s resilience and capacity for effort, as well as their affection.

### 3.10. Future Perspectives

With regard to how participants anticipate their family’s future, the results show that they try to focus on a dimension of hope and resilience, emphasizing perseverance, growth and strength: “God give me strength to keep fighting and give my daughter the maximum quality of life” (N., father of an 11-year-old child with an undiagnosed etiological disease). It should be noted that only one participant highlighted the importance of preparation for death, relative to her anticipatory grief process.

Most of the participants made suggestions to promote their quality of life—namely, psychological support for the family and resources for parents, such as access to relevant information, a network of alternative caregivers, the adaptation of the labour market and professionals with specific training to work in the context of paediatric palliative care.

## 4. Discussion

The moment of diagnosis of the illness may bring some relief, as it results in knowledge of the situation and an understanding of the complaints of the child/youth. However, this is followed by a period of emotional turmoil, as there is a psychological disorganization which is inherent to an unexpected, impactful and potentially traumatic life event [21,55]. In this context, the honesty and clarity of communication from a multidisciplinary team is essential for families, since the way the diagnosis is conveyed influences the parents’ perception of the illness, with an impact on the parents’ management of hope, coping and well-being [56].

The results of this study are congruent with the international findings, which highlight the importance of the family being properly informed about the illness and easing the burden of related words [23], as there are expressions strongly associated with the imminence of danger and death which increase anxiety in the family [56]. In fact, communication between the family and health professionals is therefore fundamental and constitutes one of the main challenges in the context of paediatric palliative care [57].

Regarding their emotional management and expression, the parent caregivers emphasize the constant worries about the illness, the routine, the child/youth, the family and the provision of care, which is in line with the literature [58]. In the scope of the future perspectives and the significant ambivalence between the fear of death and hope for healing, the anticipatory grief process is an important dimension and represents the main concern of parent caregivers. Additionally, it is common to have the feeling of not being understood and/or supported in this process [59]. The literature has shown that for these families, the management of others’ lack of understanding regarding their care and attention routines with the sick child/youth is one of the main challenges that they face [60]. For this reason, psychological support for family members is indispensable, especially through peer groups.

In fact, it is essential to address the holistic needs of each family, understanding that the physical and psychological care of the child/youth is closely linked to the psychological and social well-being of all the members of the family system [57]. Parent caregivers tend to feel overburdened [61] and refer to the ambivalence of the importance and need for self-care, on the one hand, and the difficulty in creating these moments as a result of having to care for the sick child/youth, on the other [62,63]. Within this scope, they also mentioned the deprivation of leisure and social opportunities [20] and the lack of support, inclusion, information and awareness in society [60].

Nevertheless, this study presents interesting results in line with the national literature in this area, which has highlighted the value of caregivers’ involvement in hospital dynamics as a whole—namely, in the participation in care, in cooperation with the multidisciplinary team and in the access to relevant and effective information [64,65].

During their process of adaptation to the illness, parents try to preserve the perception of their sense of competence as a parental caregiver in managing parental stress and the constant efforts towards improving their quality of life [32]. For these reasons, they implement coping strategies that can be cognitive [43,66], emotional [67,68], behavioural [36,67], relational [34,57] and spiritual [35]. The results of the present study are congruent with the research of Smith et al., who highlight the main needs of parents at the level of their hope, specifically: organizing basic needs, connecting with other people, prioritizing self-care, obtaining meaningful information, living one day at a time, participating in care and decisions, expressing positivity, and celebrating small achievements [67].

In addition to this, the way parent caregivers represent and characterize their sick child is essential to how they will manage and adapt throughout the process of the illness. The results of this study, in which parents focus on the functional limitations caused by the illness to the child [23], reflect the need to promote greater perception of the positive characteristics in children with a complex chronic illness, based on their physical and psychological qualities, which seems to help reduce the perceived magnitude of their physical disability resulting from the illness [69].

From a systemic point of view, the complex chronic disease in the paediatric age is also reflected in some changes experienced at the family level—namely, in its structure, dynamics, communication, subsystems, skills and processes [34], especially due to the demands of care provision [70]. This estrangement may also exist in relation to healthy children, since the care for the child who is ill is redoubled and the attention and care for the former is decreased [38,71]. The impact on the couple is also important—namely, through the mutual support of the spouses or, on the contrary, the deprivation of marital time [34].

In fact, greater social support has been related to post-traumatic growth [45]—namely, the skills associated with the identification of positive psychological changes during the process of adaptation to the illness [59]. Laufer and Isman investigated post-traumatic growth among 257 parents of children with special needs and had similar results to this study, concluding that this was reported by more than half of them. The authors also concluded that 74% of the caregivers reported changes in life appreciation, 69% in personal growth and 39% in the spiritual domains [72]. The results are corroborated by another study, in which parent caregivers valued life more and felt gratitude and were more sensitive to the needs of others [38].

The present study has some limitations: first, the small and non-probabilistic sample size due to the difficulty in accessing this population; second, the lack of representativeness of the sample and the presence of only one father do not allow for us to generalize the results; third, the diversity of complex chronic diseases that were included in the sample and heterogeneity in the age of the sick children and young people can contribute to the dispersion of the results found; and fourth, this study was developed during the COVID-19 pandemic and contact with the participants was exclusively online, which may have influenced the findings.

## 5. Conclusions

This research sheds light upon the resources and strategies that activate key processes in family resilience, thus enabling their promotion in the context of illness. This study emphasizes the importance of addressing the holistic needs of families, promoting effective communication with health professionals, having opportunities to express emotions, enhancing the perception of positive characteristics in children with palliative needs, creating time for the couple and sharing psychoeducation about anxiety management and self-care practices.

Within the scope of perspectives for psychological intervention in this context, the participation of these parents in leisure and rest moments is suggested, as well as the support in managing anxiety and fatigue, the involvement of the different members of the family system, the enhancement of the marital dyad and co-parenting and the reinforcement of attention in families whose children and youths depend on respiratory medical technology.

This study seeks to pave the way for new lines of research, such as exploring the specific impact of hospitalizations, communication with health professionals, decision-making and fathers’ experience. It would also be important to deepen the knowledge regarding protective factors in the adaptation to illness using longitudinal studies and larger samples.

## Data Availability

The data that supports the findings of this study are available on request from the corresponding author. The data are not publicly available as they contain information that could compromise the privacy of research participants.
